# Historical perspective on the synonymization of the four major pest species belonging to the *Bactrocera dorsalis* species complex (Diptera, Tephritidae)

**DOI:** 10.3897/zookeys.540.6028

**Published:** 2015-11-26

**Authors:** Alvin K.W. Hee, Suk-Ling Wee, Ritsuo Nishida, Hajime Ono, Jorge Hendrichs, David S. Haymer, Keng-Hong Tan

**Affiliations:** 1 Department of Biology, Faculty of Science, Universiti Putra Malaysia, 43400 UPM Serdang, Selangor Darul Ehsan, Malaysia; 2 School of Environmental and Natural Resource Sciences,; 3 Centre of Insect Systematics, Faculty of Science and Technology, Universiti Kebangsaan Malaysia, Bangi, Malaysia; 4 Laboratory of Chemical Ecology, Kyoto University, Kyoto, Japan; 5 FAO/IAEA Joint Division of Nuclear Techniques in Food and Agriculture, Vienna, Austria; 6 Department of Cell and Molecular Biology, University of Hawaii, Honolulu, HI, United States of America; 7 Tan Hak Heng Co., Penang, Malaysia

**Keywords:** *Bactrocera dorsalis* species complex, *Bactrocera philippinensis*, *Bactrocera papayae*, *Bactrocera invadens*, synonymization, integrative taxonomy

## Abstract

An FAO/IAEA-sponsored coordinated research project on integrative taxonomy, involving close to 50 researchers from at least 20 countries, culminated in a significant breakthrough in the recognition that four major pest species, *Bactrocera dorsalis*, *Bactrocera philippinensis*, *Bactrocera papayae* and *Bactrocera invadens*, belong to the same biological species, *Bactrocera dorsalis*. The successful conclusion of this initiative is expected to significantly facilitate global agricultural trade, primarily through the lifting of quarantine restrictions that have long affected many countries, especially those in regions such as Asia and Africa that have large potential for fresh fruit and vegetable commodity exports. This work stems from two taxonomic studies: a revision in 1994 that significantly increased the number of described species in the *Bactrocera dorsalis* species complex; and the description in 2005 of *Bactrocera invadens*, then newly incursive in Africa. While taxonomically valid species, many biologists considered that these were different names for one biological species. Many disagreements confounded attempts to develop a solution for resolving this taxonomic issue, before the FAO/IAEA project commenced. Crucial to understanding the success of that initiative is an accounting of the historical events and perspectives leading up to the international, multidisciplinary collaborative efforts that successfully achieved the final synonymization. This review highlights the 21 year journey taken to achieve this outcome.

## Introduction

The genus *Bactrocera* Macquart of true fruit flies belongs to the Dacinae - a subfamily of Tephritidae (Drew 1989). Over 500 species have been described as belonging to this genus, making it one of the largest genera within the Tephritidae (Drew 1989). It has been estimated that total damage caused by tephritid fruit flies affecting production, harvesting, packing, and marketing of fruits globally amounts to over US$2 billion annually (Shelly et al. 2014).

Within *Bactrocera*, the *Bactrocera dorsalis* species complex contains almost 100 species that are morphologically similar and occur in the tropics and subtropics where fruit growing areas are extensive (Drew and Romig 2013). A number of species in this complex are of economic importance and highly invasive, the best known of which is the Oriental fruit fly, *Bactrocera dorsalis* (Hendel). Closely linked in pest status with *Bactrocera dorsalis* are the now synonymized species *Bactrocera papayae* Drew & Hancock, *Bactrocera philippinensis* Drew & Hancock and *Bactrocera invadens* Drew, Tsuruta & White. *Bactrocera dorsalis* is known to cause devastating losses in fruit commodities, especially in the Asia-Pacific and Africa regions (Kawasaki et al. 1991, Ye 2001, Verghese et al. 2004, Clarke et al 2005, De Meyer et al. 2010, Khamis et al. 2012, Li et al. 2012).

The existence of a complex of closely related, morphologically similar species to *Bactrocera dorsalis*, had been recognized for over 40 years (Hardy and Adachi 1954) before a major taxonomic revision of the group was undertaken by Drew and Hancock (1994). This revision described over 50 new species in the complex and, while the biological validity of most described species has not been questioned, this has not been the case for all taxa, particularly some of the very important pest species such as *Bactrocera papayae*. For these pest species, serious questions have been raised as to the validity over their status as separate species. This issue was accentuated when Drew et al. (2005) described *Bactrocera invadens* from Africa, a new species which again could not be consistently and readily separated from *Bactrocera dorsalis*.

The extensive similarities between *Bactrocera dorsalis* *s.s* and the three putative species established, *Bactrocera invadens*, *Bactrocera papayae* and *Bactrocera philippinensis*, has led to much debate on the delimitation of these species, particularly in terms of seeking congruence between the biological and taxonomic status of these entities (Tan 2003, Clarke et al. 2005, Drew et al. 2008). Numerous studies over the past two decades have contributed to our understanding of the pest species in the *Bactrocera dorsalis* complex particularly with respect to their morphological and biological attributes. Nonetheless, these studies had not reduced the complexities of the species’ status, as evident from a rise in the number of species in the *Bactrocera dorsalis* complex to almost 100 in the years since the 1994 revision in (Drew and Romig 2013).

To address and resolve the longstanding issue of species delimitation in the key pest species of the *Bactrocera dorsalis* complex, a multidisciplinary approach was adopted by an international team of more than fifty researchers from over twenty different countries. Under the auspices of the Joint FAO/IAEA Division on Nuclear Techniques in Food and Agriculture, a 5-year Coordinated Research Project (CRP) on *‘Resolution of Cryptic Species Complexes of Tephritid Pests to Overcome Constraints to SIT Application and International Trade’* was established in 2010. The aim of the project was to define the species limits of pest species complexes within the Tephritidae, with the *Bactrocera dorsalis* complex identified as a priority. Studies that had been independently developed in the past, including morphometric, cytogenetic, molecular, behavioural and chemoecological datasets were re-examined, and gaps crucial for answering questions of how taxonomic species could be reconciled as biological species were filled. This project has led to the synonymization of *Bactrocera papayae*, *Bactrocera philippinensis* and *Bactrocera invadens* with *Bactrocera dorsalis*, based on the conclusion that there is insufficient evidence to maintain the former three taxa as biological species distinct from *Bactrocera dorsalis* (Schutze et al. 2015a).

It is not the aim of this paper to again provide the evidence for the synonymization of the four major pest species, as this has already been provided (Schutze et al. 2015a). Rather, it is our intent here to ensure that the long and arduous journey taken to achieve this outcome is understood. We feel that it is vitally important that the younger generation of fruit fly workers, who though armed with advanced scientific skills and techniques, will appreciate the background and good science conducted from the beginning in resolving this issue of significant transboundary importance for international agricultural trade. Thus, this paper aims to provide a historical account of the events leading to the FAO/IAEA-sponsored international efforts in resolving this prickly issue.

## Taxonomic history of *Bactrocera dorsalis* complex

Before describing some of the biological insights which led to the questioning of taxonomic validity of these species, this section details the taxonomic history of the taxa of concern.

### *Bactrocera papayae*, *Bactrocera carambolae* and *Bactrocera philippinensis*

Prior to the taxonomic revision of the *Bactrocera dorsalis* complex by Drew and Hancock (1994), the taxa endemic to the southeast Asian region of Malaysia, Indonesia and Thailand were identified as a single species, viz. *Bactrocera* (= *Dacus*) *dorsalis* (Hendel) (Hardy and Adachi 1954, Tan and Lee 1982). A second taxon was subsequently recognized, being referred to before description as Malaysian B (Drew 1991) and ‘sp. near *Bactrocera dorsalis* (B)’ (White and Elson-Harris 1992), before being formally described as *Bactrocera papayae* Drew and Hancock (1994). This taxon was given the common name of Asian papaya fly (Drew 1997), although studies in Malaysia had shown that papaya was not the preferred host of this species, which prefers instead starfruit and banana (Tan and Nishida 1996). The detection of *Bactrocera papayae* in northern Queensland, Australia, in 1995 resulted in a successful eradication programme costing over US$32.5 million (Fay et al. 1997, Cantrell et al. 2002).

The concern over the destructive potential of *Bactrocera papayae* also underscored the importance of another closely related species, *Bactrocera carambolae* Drew & Hancock, which itself had formerly been referred to as Malaysian A (Drew 1991) and sp. near *Bactrocera dorsalis* (A) (White and Elson-Harris 1992) and was found together with *Bactrocera papayae* in Peninsular Malaysia and southern Thailand (Clarke et al. 2001). Together, *Bactrocera papayae* and *Bactrocera carambolae* accounted for the most damage to fresh fruits in Malaysia. These species were already known to be morphologically similar and able to interbreed resulting in viable laboratory offspring with hybrid rectal pheromonal compounds, even up to the F_3_ generation (Wee 2000). Natural hybrids of both *Bactrocera carambolae* and *Bactrocera papayae* possessing similar rectal pheromonal compounds to those of laboratory hybrids had also been detected from the field (Wee and Tan 2005).

Additional to ‘species near *Bactrocera dorsalis* (A) and (B), was a third taxon, designated as ‘sp. near *Bactrocera dorsalis* (C) (White and Elson-Harris 1992). This population was only known from the Philippines and was subsequently described by Drew and Hancock (1994) as *Bactrocera philippinensis*.

### Bactrocera invadens

As for the South-east Asian pest species of the *Bactrocera dorsalis* complex, confusion also existed for *Bactrocera invadens* in Africa, a devastating pest species now widespread in Africa, which has largely displaced other long established pests such as Mediterranean fruit fly, *Ceratitis capitata* (Wiedemann) (Hill and Treblanche 2014). Between its first detection in Kenya, 2003 and subsequent description as a new species (Drew et al. 2005), it was believed to originate from the *Bactrocera dorsalis* group, and was most likely *Bactrocera dorsalis* s.s. (Lux et al. 2003). Drew et al. (2005) and Drew et al. (2008) treated *Bactrocera invadens* as part of the *Bactrocera dorsalis* complex, but it was subsequently removed from the complex (Drew and Romig 2013).

## Accumulating evidence for synonymisation – 1994 to 2010

### Biological insights from chemical ecology

Efforts of fruit fly workers over two decades to resolve the biological species status of *Bactrocera papayae*, *Bactrocera philippinensis*, *Bactrocera invadens* and *Bactrocera dorsalis* started with the question from one of us (Keng-Hong Tan) on why males of certain fly species, such as *Bactrocera dorsalis*, are so strongly attracted to methyl eugenol (ME). This research effort was partly in response to a challenge posed by N. Tanaka of the USDA Hawaiian Fruit Flies Investigations Laboratory in 1980 to figure out the role of ME in the biology of male tephritid flies. Whilst ME was first discovered a century ago as attracting male fruit flies (Howlett 1915), it was not until 60 years later that it was shown to be a highly potent attractant for *Bactrocera dorsalis* (Metcalf et al. 1975). While known to respond to ME, the question of why male flies are uniquely attracted to ME has been considered as a great mystery of tephritid biology (Cunningham 1989). ME is found as a common phenylpropanoid in numerous species of plants (>450 species in 80 families of plants) (Tan and Nishida 2012); and synthetic ME has been successfully used for monitoring and control programmes, including area-wide and border surveillance, quarantine and male annihilation programmes worldwide against many ME-responding tephritid pests of major economic importance (see reviews in Vargas et al. 2010; Shelly et al. 2014, Tan et al. 2014).

While Tan and colleagues searched for the reason as to why male *Bactrocera* respond to ME, significant insights were also gained as to the likely biological relationships between *Bactrocera dorsalis* and *Bactrocera papayae* (especially). This section describes the history of that process.

In the early 1980s work was undertaken using ME for field ecological surveys and population dynamics of *Dacus* species. This work demonstrated for the first time that temporary habituation of the male flies to ME is possible (Tan and Lee 1982; Tan 1984, 1985; Tan and Jaal 1986; Tan et al. 1987; Tan and Serit 1988). Following that and concomitant with continued ecological work involving ME, there was also interest in understanding the fate of consumed ME in relation to fruit fly physiology. Initial work by Keng-Hong Tan showed that, based on thin layer chromatography (TLC) analyses of haemolymph and digestive tissues of ME-fed and ME-deprived male flies, consumed ME was detected in the crop organ only. Furthermore, two saliva spots (with different relative mobilities from ME), that resulted from ME feeding by live sexually mature males (without prior exposure to ME), were detected on the developed-TLC plate only for extracts of ME-fed male rectal gland, crop and haemolymph (least visualized). This suggested the possibility that the rectal (pheromone) gland contained some compounds derived from the crop upon feeding of ME that were carried to the gland by the haemolymph. The transport of the ME-derived compounds to the rectal gland was subsequently confirmed (Hee and Tan 2004, 2005, 2006).

In 1986, the research collaboration of Keng-Hong Tan and Ritsuo Nishida of Kyoto University on the ecological significance of male fruit fly attractants took off after the introduction of Nishida by Professor S. Takahashi also of Kyoto University to Tan. This research collaboration resulted in the detection of the phenylpropanoids, (*E*)-coniferyl alcohol (CF) and 2-allyl-4,5-dimethoxyphenol (DMP), as sex pheromone components of male *Bactrocera dorsalis* (samples from Malaysia later known as ‘Malaysian B’ and then *Bactrocera papayae* - see next paragraph) following ME consumption (Nishida et al 1988a, 1988b).

During that time, *Bactrocera dorsalis* in Malaysia had been taxonomically split into two sibling species, Malaysian A and Malaysian B in 1991, and subsequently in 1994 described as *Bactrocera carambolae* and *Bactrocera papayae*, respectively. These sibling species, together with *Bactrocera dorsalis* *s.s.*, were morphologically very similar to each other, particularly for male flies, and much work was concentrated on attempts to differentiate these species. In 1994, results from a primary chemotaxonomic diagnostic tool were presented in the 4^th^ International Symposium of Fruit Flies of Economic Importance held in Tampa, Florida, demonstrating that CF and DMP were detected in both *Bactrocera dorsalis* (ex-Hawaii) and *Bactrocera papayae*, and with improved male mating competitiveness, following males’ consumption of ME (Tan and Nishida 1996). Both the compounds were also shown to be male attractants, although CF was more attractive than DMP to conspecific females (Khoo et al. 2000). At the point source of highest concentration of these chemicals, females were observed to extrude their ovipositor, a sign of mating acceptance, confirming their role in mating behavior (Tan and Nishida 1996, Hee and Tan 1998). This information revealed the role of ME as a precursor to the ME-derived male sex pheromone components, which might be the main reason for the high mating percentages observed between the two sibling species in an outdoor cage (Tan and Nishida 1996).

The discovery that both *Bactrocera dorsalis* and *Bactrocera papayae*, having very similar morphological characters, also possessed identical pheromone components and mated readily under semi-natural conditions and producing viable offspring over generations provided the first strong evidence that the two species deserved to be considered as a single biological species. These are the basic criteria for species delimitation of Mayr’s ‘Biological Species Concept’ (Mayr 1957).

### Biochemical and genetic analyses

Following the revision of the *Bactrocera dorsalis* complex in 1994, differences in male genitalia length was used as the sole morphological basis of separating *Bactrocera papayae* from *Bactrocera dorsalis* (Drew and Hancock 1994; but see also Iwahashi 2001). Isozyme and other genetic evaluation of the two species were also conducted in an effort to provide evidence for species discrimination. However, while a discrimination was deemed possible, both species were shown to possess close genetic affinity (Yong 1994), including the lack of species-specific alleles and loci (Yong 1995). In general isozymes are now known to not be suitable markers to differentiate species due to factors such as hidden genetic variation (Behura 2006).

The development of DNA-based markers meant that a broader range of molecular tools were available to researchers in clarifying the status of species. Using ribosomal DNA markers, for example, had shown in tephritids that it was possible to differentiate distinct species within and between genera of flies, but not within a complex of closely related species (Armstrong and Cameron 2000). For example within the *Bactrocera dorsalis* complex, no ribosomal markers in this region could be found to distinguish between *Bactrocera dorsalis*, *Bactrocera papayae* and *Bactrocera philippinensis*. In addition, another investigation that included an additional sympatric sibling species, *Bactrocera carambolae*, showed that this species also could not be discriminated by phylogenetic analyses using mitochondrial sequences (Muraji and Nakahara 2001). In this paper, the authors noted *“Although the monophylies of most of the species were supported by both topologies and bootstrap analyses, the two individuals of Bactrocera papayae did not form a clade for a species. Bactrocera papayae and its allied insects were formerly considered as a single species, Bactrocera dorsalis (Hendel)”*. The authors further stated *“Thus, Bactrocera papayae samples used in this study may be hybrids or interbreeding descendants, which might explain why the nucleotide sequences did not match the species identification based on morphological characteristics”*. In addition, Smith et al. (2003) also showed that of the four sibling species in the *Bactrocera dorsalis* complex that included *Bactrocera carambolae* and *Bactrocera caryeae*, both *Bactrocera dorsalis* and *Bactrocera papayae* belonged to the same clade within the 24 *Bactrocera* species analysed. Based on a phylogenetic species theory, this suggested that *Bactrocera dorsalis* and *Bactrocera papayae* were not distinct species.

Additional molecular work on species in the *Bactrocera dorsalis* complex began with the analysis of the actin gene family in *Bactrocera dorsalis* (He and Haymer 1994). Specifically, intron sequences from these genes were used for the analysis of genetic variation in populations of *Bactrocera dorsalis* and its sibling species in the *Bactrocera dorsalis* complex (He and Haymer 1997, 2003). Introns were used primarily because, as noncoding sequences, they were typically known to be much more variable than conserved coding sequences. Because of this, it was presumed that intron sequences could serve to identify genetic markers useful for differentiating species and populations. The variable intron markers identified were also used to construct oligonucleotide arrays for rapid screening of genetic variation in populations of *Bactrocera dorsalis*, *Bactrocera papayae* and *Bactrocera carambolae* (Naeole and Haymer 2003). In this work, Naeole and Haymer showed that one of three actin alleles were identical in DNA sequence and therefore shared between *Bactrocera dorsalis* and *Bactrocera papayae*. They speculated that this result was more consistent with these taxa representing populations of the same species, rather than being distinct species. Further discussions on this possibility, as reported by Tan (2003), allowed for the beginnings of a comprehensive picture to emerge incorporating support for this idea from a wide range of datasets.

Following this, samples of *Bactrocera* flies were sent to Alfred Handler (USDA, Gainesville, Florida) from Keng-Hong Tan. The analysis of insertions of the *piggyBac* transposable element in these specimens revealed that the gene was inserted at identical loci in both *Bactrocera dorsalis* and *Bactrocera papayae*, but at different loci in other clearly distinct species (Handler et al. 2008). During the 9^th^ Exotic Fruit Fly Symposium in Fresno, California in 2007, Handler confirmed to Tan (personal communication) that this result would be expected to occur only with extremely closely related taxa, such as between different strains of the same species that can easily interbreed and produce viable offspring, as opposed to entities representing distinct species. As a matter-of-fact, using this same approach, Zimowska and Handler (2005) had earlier found no significant difference between that of *Bactrocera dorsalis* and *Bactrocera papayae*.

### Absence of post-zygotic reproductive isolation between *Bactrocera dorsalis* and *Bactrocera papayae*

That *Bactrocera dorsalis* and *Bactrocera papayae* were not distinct species was consistent with earlier observations that interbreeding between *Bactrocera papayae*, *Bactrocera carambolae* and *Bactrocera dorsalis* results in viable offspring/hybrids (unpublished data c/f Tan 2000). Further, studies in Malaysia showed that mating compatibility between *Bactrocera dorsalis*, *Bactrocera papayae* and *Bactrocera carambolae* increased following males’ consumption of ME (Wee and Tan 2000; Tan 2003). In these cases, the production of an identical phenylpropanoid, CF, functioning as a male sex pheromone component in males of those species, was suggested to be a factor in explaining the enhanced interbreeding seen in males that had consumed ME (Tan and Nishida 1996, Tan 2000). Furthermore no post-zygotic isolation was observed in matings between *Bactrocera dorsalis* (ex-Hawaii) and *Bactrocera papayae*, resulted even in F_3_ hybrid offspring in the laboratory (Tan 2003).

While some authors have refuted this line of argument supporting the view that *Bactrocera dorsalis* and *Bactrocera papayae* are the same species, noting that hybridization between *Bactrocera* species is easy to achieve in laboratory cages even when using species from different subgenera (Cruickshank et al. 2001; Drew et al. 2008), interspecific matings between sibling species of *Bactrocera dorsalis*, *Bactrocera papayae* and *Bactrocera carambolae* have been reported to occur in field (McInnis et al. 1999; Wee and Tan 2000). Furthermore, in field studies, using modified ME-baited clear traps without a toxicant, the occurrence of natural hybridization between the sympatric sibling species, *Bactrocera papayae* and *Bactrocera carambolae* has been supported by captures of wild hybrids and chemotaxonomy (Wee and Tan 2005).

### Persistence in maintaining the validity of *Bactrocera dorsalis* and *Bactrocera papayae* as distinct species

The failure to discover robust diagnostics markers to separate the species within the complex, and indeed the accumulating evidence that supported the idea that at least *Bactrocera dorsalis* and *Bactrocera papayae* were populations of the same species, prompted further studies by Drew et al. (2008) using additional morphometric measurements in examining the taxonomic status of a number of species in the *Bactrocera dorsalis* complex including *Bactrocera dorsalis* and *Bactrocera papayae*. In that paper, the authors argued that the accumulated evidence, including data based on morphometrics, pheromones, allozyme and nucleotide studies still supported maintaining these taxa as distinct species, even in light of the information presented in the preceding paragraphs that strongly suggested that at least *Bactrocera dorsalis* and *Bactrocera papayae* should be considered a single species. Further, whilst a review by Clarke et al. (2005) highlighted the difficulties in using morphological criteria to distinguish between those species in the *Bactrocera dorsalis* complex, particularly that of *Bactrocera dorsalis* and *Bactrocera papayae*, the use of certain genetic diagnostic tools in the discrimination of those two species was then generally accepted. For the evidence at that time, as to why Clarke et al. and Drew et al. argued the species were valid, the reader is directed to those papers. In retrospect, and despite the strengths and weaknesses of the many individual studies and their interpretations, it seems that at the time a fully integrative taxonomic study might have contributed to resolving this issue by asking whether species that can be separated only morphologically can, in fact, be said to represent valid biological species (Clarke and Schutze 2014).

As soon as the paper by Drew et al. (2008) was published, Tan was alerted by Jorge Hendrichs of the FAO/IAEA of the authors’ arguments in support of the idea that *Bactrocera dorsalis* and *Bactrocera papayae* were in fact distinct biological species. This paper included the statement that *“Although genetic similarities between allopatric populations of Bactrocera dorsalis and Bactrocera papayae have been documented (Tan, 2003), this does not cast doubt on their species status”*. It is believed that the game changer to this issue was the sharp response of Keng-Hong Tan (in a form of a 4-page rebuttal letter sent via email to the journal editor) in relation to this article. In his letter, Tan pointed out that regrettably, the authors of the paper chose to ignore the increasing evidence clearly showing that the two taxa deserved recognition as one biological species. Instead, as the core focus of the article, they insisted that they were valid species based largely on morphological evaluation. The rebuttal letter was also informally sent to fruit fly researchers and members of FAO/IAEA and the International Plant Protection Convention Fruit Fly Technical Panel. Taking cognizance of the facts pointing to both *Bactrocera papayae* and *Bactrocera dorsalis* as indistinct species, we (Keng-Hong Tan, Suk-Ling Wee and Alvin Kah-Wei Hee) have already been referring to both taxa as that of *Bactrocera dorsalis* as early as 2005 in our papers (Hee and Tan 2005, 2006, Wee and Tan 2007), which were several years earlier than the implementation of the FAO/IAEA-sponsored CRP.

Furthermore, it must also be pointed out that at the same time of this fierce debate, evidence was being accumulated showing the devastation caused by another species, *Bactrocera invadens* as it invaded and spread rapidly across the continent of Africa in the early 2000s, together with the establishment of quarantine barriers by countries that did not harbour *Bactrocera invadens*, even though they harboured *Bactrocera dorsalis*.

### *Bactrocera invadens* as the final impetus for coordinated international action

The detection of an unknown pest fruit fly in Kenya, 2003, first reported as an unknown species suspected to be of Asian origin and related to the Oriental fruit fly (Lux et al. 2003), struck fear in the hearts of quarantine and trade authorities. Given much was known about the destructive losses caused by the *Bactrocera dorsalis* in many countries, the incursion of a species closely related to *Bactrocera dorsalis* was of great concern as it could potentially devastate the agricultural and fruit industries that many African countries in the tropics and subtropics depend upon. The incursion was in fact swift and destructive, with over 40 species of fruits of economic importance infested in 30 African countries (Khamis et al. 2012). This had the international fruit fly scientific community, particularly workers in Africa and Europe, scrambling resources to seek more information on the fly’s basic ecology, biology and control. Described as a new species, *Bactrocera invadens*, in 2005, over 80 refereed papers have been published on this fly since (for a brief overview, see Schutze et al. 2015b).

One major reason for the need to resolve the taxonomic status of this new pest was to confirm or reject the validity of the quarantine barriers which were established following the *Bactrocera invadens* incursion between importing countries (for example in Asia) where *Bactrocera dorsalis* was endemic and exporting African nations. In addition, an effective fruit fly management programme requires correct identification of the target pest species. In the case of *Bactrocera invadens*, while copious amounts of work have been done in understanding its ecology and biology, its true taxonomic status remained confused despite the availability of an array of modern analytical tools to aid in the identification of the species. For example, using *piggyBac* gene insertions, Zimowska and Handler (2005) found that *Bactrocera invadens* was indistinguishable from *Bactrocera dorsalis* *s.s.* and *Bactrocera papayae*, but this workshop abstract was not taken on to the peer reviewed literature. It must be noted that the results of Zimowska and Handler (2005) were based on the use of fly samples obtained from Tim Holler of USDA-APHIS, just prior to the formal description of these as *Bactrocera invadens*. These samples had been collected from two different populations in Tanzania (location in and around Dar Es Salaam) in December 2003 (Handler, personal communication). At the time, much of work focused on morphological evaluation, resulting in much continuing controversy on the status of this taxon as a distinct species. As an example, one of the diagnostic distinguishing characters was the appearance of varying shades of scutum colour from pale- to red-brown and almost black in *Bactrocera invadens* (Drew et al. 2005; Drew et al. 2008). The uncertainty over the species’ status based on morphological features alone raised fears among some fruit fly workers that deliberation over the identity of *Bactrocera invadens* would set the stage for another grueling scientific argument, similar to that which occurred over the status of *Bactrocera dorsalis* and *Bactrocera papayae*.

## The Coordinated Research Project

In conjunction with the alarming spread of *Bactrocera invadens* in Africa and doubts cast over its taxonomic status, as well the ongoing failure to resolve the species status of the Asian species, a clear need and opportunity arose for the international fruit fly research community to address the question of species boundaries in the *Bactrocera dorsalis* complex, involving not only *Bactrocera invadens* but also *Bactrocera dorsalis*, *Bactrocera papayae*, *Bactrocera philippinensis* and *Bactrocera carambolae*, all of which rank among the world’s most destructive and highly invasive alien pest species. At the request from member states, the Joint FAO/IAEA Division took on an instrumental role in establishing in 2010 a six-year Coordinated Research Project (CRP) on *‘Resolution of Cryptic Species Complexes of Tephritid Flies to Overcome Constraints to SIT Application and International Trade’*. While the first meeting of researchers to coordinate the research programme of this CRP took place in Vienna in 2010, it must be noted that it was during the FAO/IAEA consultants meeting in 2009 (that included Jorge Hendrichs and Keng-Hong Tan) that the CRP was designed.

The objectives of the CRP were not only to resolve the species issues within the *Bactrocera dorsalis* complex but also of other tephritid genera where close species relationships were an issue, including *Anastrepha* and *Ceratitis* pest populations or species. Research coordination meetings were held in Vienna, Austria (2010), Brisbane, Australia, (2012), Tucuman, Argentina (2013), and La Reunion, France (2015), where research progress was critically evaluated and follow-up research work-plans developed. A multidisciplinary consensus was finally reached that while there was sufficient evidence confirming the separate species status for *Bactrocera carambolae*, the four species *Bactrocera dorsalis*, *Bactrocera invadens*, *Bactrocera papayae* and *Bactrocera philippinensis* constituted only a single biological species. This culminated in the synonymization of the later three species with *Bactrocera dorsalis* (Schutze et al. 2015a). The large authorship of the paper (49 authors from 15 countries) attests to the commitment, dedication and involvement of the international community to resolving this issue. As this outcome is celebrated, it must be remembered that doing good science through perseverance is not the only prerequisite for success, but strong scientific leadership in adopting the philosophy that asking and pursuing answers to the right questions is of paramount importance. Finally, we acknowledge that more research needs to be conducted to fill gaps in the biological, ecological and evolutionary understanding of other sibling species within the *Bactrocera dorsalis* complex, particularly incipient species in the Asia-Pacific region.
